# Systematic analysis of the pharmacogenomics landscape towards clinical implementation of precision therapeutics in Greece

**DOI:** 10.1186/s40246-025-00720-1

**Published:** 2025-02-07

**Authors:** George P. Patrinos, Kariofyllis Karamperis, Margarita-Ioanna Koufaki, Maria Skokou, Zoe Kordou, Eirini Sparaki, Margarita Skaraki, Christina Mitropoulou

**Affiliations:** 1 Department of Pharmacy, Laboratory of Pharmacogenomics and Individualized Therapy, University of Patras School of Health Sciences, University Campus, Rion, Patras, GR-265 04 Greece; 2 College of Medicine and Health Sciences, Department of Genetics and Genomics, United Arab Emirates University, Al-Ain, Abu Dhabi, United Arab Emirates; 3 Zayed Center for Health Sciences, United Arab Emirates University, Al-Ain, Abu Dhabi, United Arab Emirates; 4 Faculty of Medicine and Health Sciences, Department of Pathology, Clinical Bioinformatics Unit, Erasmus University Medical Center, Rotterdam, the Netherlands; 5 The Golden Helix Foundation, London, UK

**Keywords:** Pharmacogenomics, Personalized medicine, Clinical implementation, Service providers, Regulatory guidance, Precision therapeutics

## Abstract

Pharmacogenomics (PGx) aims to delineate a patient’s genetic profile with differences in drug efficacy and/or toxicity, particularly focusing on genes encoding for drug-metabolizing enzymes and transporters. Clinical implementation of PGx is a complex undertaking involving a multidisciplinary approach that includes, among others, a thorough understanding of a country’s preparedness to adopt this modern discipline and a detailed knowledge of PGx biomarkers allelic spectrum at a population level. In several European populations, particularly in countries with lower income, clinical implementation of PGx is still in its infancy. We have previously performed a pilot study to determine the prevalence of PGx biomarkers in 18 European populations, as the first step towards population PGx at the European level. Here, we provide a comprehensive analysis of the current state of PGx in Greece, including a detailed allelic frequency spectrum of clinically actionable PGx biomarkers, the level of PGx education in academia, the provision of PGx testing services from public and private laboratories, and the aspects of the regulatory PGx environment, especially with respect to the discrepancies between the Greek National Organization of Medicines and the European Medicine Agency and health technology assessment. This study would not only provide the foundations for expediting the adoption of PGx in clinical reality in Greece but can also serve as a paradigm for replicating future studies in other European countries, to expand on previously available pilot studies.

## Introduction

Pharmacogenomics (PGx) aims to correlate differences in individuals’ genetic profiles with interindividual variability in drug use, touching both upon drug efficacy and toxicity [reviewed in 1]. A growing body of scientific evidence suggests that variants in genes encoding for drug-metabolizing enzymes and transporters directly impact their function, which in turn affects drug efficacy and/or increases the chances of developing adverse drug reactions (ADRs). As a result, a significant proportion of patients will likely go untreated or, worse, develop mild, moderate, or even serious and life-threatening ADRs for many different medications [2]. Indeed, there are more than 350 of the most commonly prescribed medications that bear PGx information in their summaries of product characteristics (SmPCs), documented in the main regulatory bodies, namely the United States Food and Drug Administration (FDA; www.fda.gov) and the European Medicines Agency (EMA; www.ema.europa.eu), hence correlating genomic biomarkers over drug dosing, safety risk, and/or efficacy [3–5].

Furthermore, there are substantial differences in the prevalence of several ADRs and medication efficacy among different populations, as a result of reciprocal differences in the prevalence of PGx variants [6]. This is described as population PGx. This emerging trend is supported by several previous studies that have revealed differences in the PGx variants’ allelic frequencies among different European [7], Southeast Asian [8], Middle Eastern [9], and Latin American populations [10]. The differences are not only limited to genetics but are also pinpointed in the preparedness of each country to implement PGx in the clinic setting, deducted from different levels of PGx education and awareness, the regulatory deficiencies for genome-guided therapeutics, the lack of such interventions’ reimbursement and so on [11].

We have previously performed a pilot study to comprehensively sketch a pan-European PGx biomarkers spectrum, by analyzing clinically relevant PGx biomarkers in 18 European populations, reporting significant population-specific differences in the prevalence of clinically actionable PGx biomarkers [7]. Here, we have performed a comprehensive population PGx analysis in Greece, as part of the Genome of Greece nationwide genomics research initiative [12] that not only involves genotyping of 46 clinically actionable PGx biomarkers in 12 pharmacogenes [13] but also a thorough analysis of the PGx landscape in the country, including PGx education in academic institutions in Greece, the existing public and private genetic laboratories that are involved in PGx testing in Greece, the regulatory framework for genome-guided therapeutics, including the level of compliance between the Greek National Organization for Medicines (NOM) and the EMA and the reimbursement of genome-guided therapeutic interventions in Greece. These data would not only pave the path for implementing PGx in clinical settings in Greece but can also serve as a paradigm for the replication of similar studies in other populations, based on previously conducted pilot studies, to soon make PGx a clinical reality worldwide.

## Methods

### Sample collection, genotyping, and statistical analysis

A total of 1269 adult subjects were included in this study for the calculation of PGx variant allele frequencies and metabolizer phenotypic status. The research described in this study has been approved by the Institutional Review Board (IRB) of the University of Patras (825 − 28/12/2016) and written informed consent was obtained from all participants before sample collection.

DNA extraction from peripheral blood or saliva and genotyping using a panel of 46 clinically actionable PGx biomarkers in 12 pharmacogenes has been performed as previously described in [14]. Differences in the prevalence of the clinically actionable PGx biomarkers in the population of Greece were compared with the European average, deducted from the 1000 genomes project (European_1000G; hg39), the Clinical Pharmacogenetics Implementation Consortium (European_CPIC; www.cpicpgx.org), the literature (European_L) and the Finnish population. Excel was used as a statistical tool. Descriptive analysis, graphs and figures were included in the data analysis and were conducted using Excel.

### Collecting information for private and public PGx service providers and PGx education in Greece

Information about the private and public genetic laboratories that offer PGx testing services in Greece was manually queried on the Internet using keywords. The names and nature of PGx testing services of these providers are available upon request. Information was also compared with our previous analyses [15].

A manual search of the curricula of the various departments of health and life sciences in Greece was conducted to review the available PGx courses and modules both at the undergraduate and postgraduate level in Greece. All curricula were compared against our previous findings [16]. Keywords used were “Pharmacogenomics”, “Pharmacogenetics”, “Personalized Medicine”, “Precision Medicine”, “Genomic Medicine”, and “Molecular Genetics”. The search was conducted in May 2024 including only Biomedical departments of Greek Universities, involving undergraduate and graduate programs as well as interdepartmental and inter-institutional graduate programs.

### Revealing and querying PGx regulatory guidance in drug labels

To identify the regulatory guidance for PGx-guided therapeutics in drug labels in Greece, we first downloaded the drug labels of all EMA (www.ema.europa.eu) approved medications, since the Greek NOM (www.eof.gr) is an EMA member. Subsequently, we gathered their SmPCs from the official site of the Greek NOM and compared it to that list of EMA. Due to the fact that all SmPCs are available in Greek, an extensive search at all available data points were accounted. For instance, we relied on the Galinos drug repository (www.galinos.gr), a database that includes SmPCs from medications that are available in the Greek market, the Greek national regulatory body website and lastly the official Greek translation of the original SmPCs as documented in the EMA website. All information was gathered and manually curated to unravel any discrepancies between the Greek NOM and the EMA for the different SmPCs. Then, a list with the source of SmPCs was created to identify which source was the most detailed or appropriate to use and sort out any duplicates. Finally, any information found about the medications with PGx guidelines was further assessed and verified using the PharmGKB database (www.pharmgkb.org).

## Results

### PGx biomarkers allelic frequencies and metabolizer status in the Greek population

**Fig. 1:**
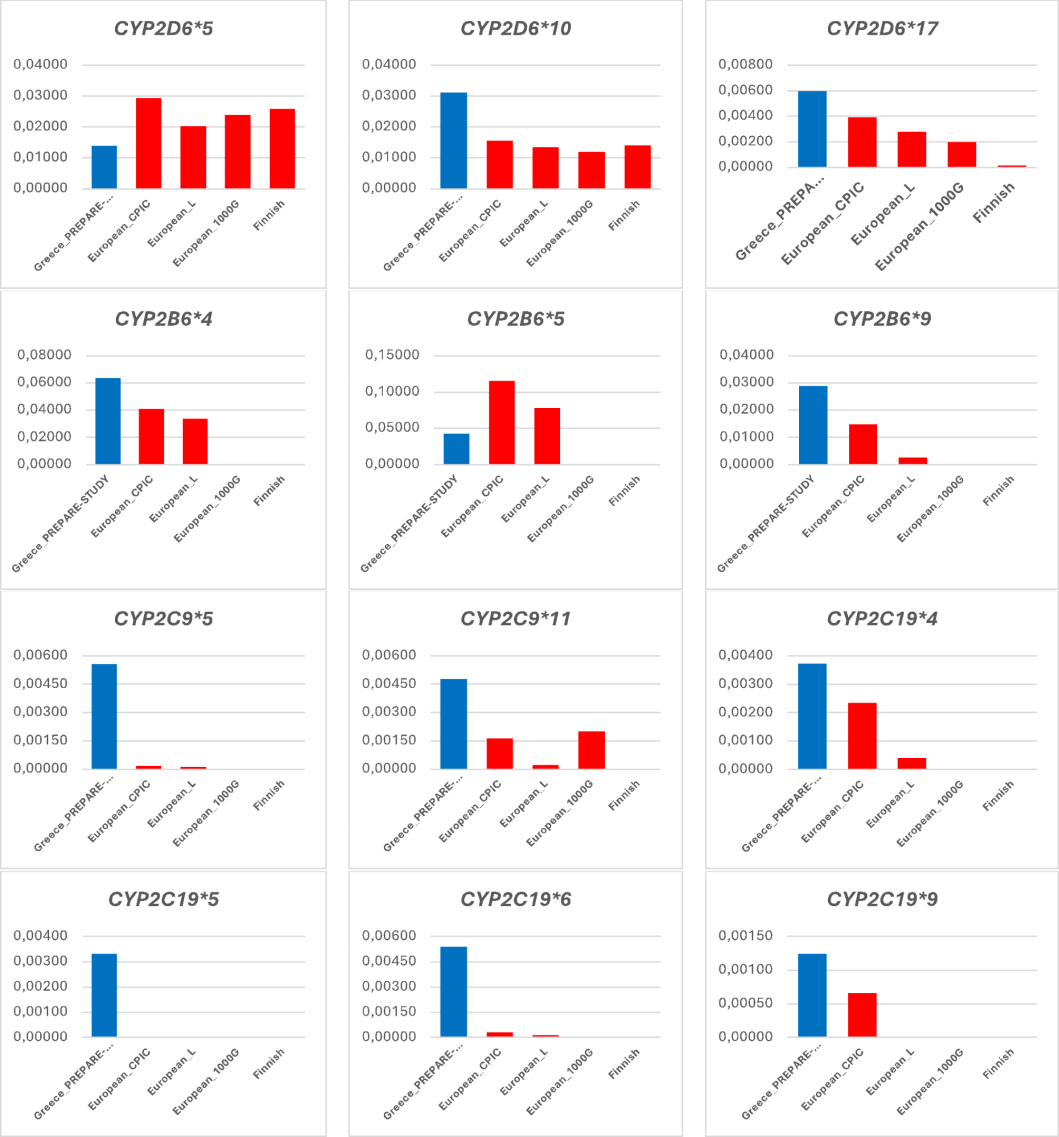
Prevalence of PGx biomarkers in the pharmacogenes in the CYP450 family in the Greek population (in blue), compared to the European average, as documented in the CPIC database, the 1000 Genomes project, the literature, and the Finnish population (in red). Abbreviations: European_L: European population data extracted from the literature, European_1000G: European population data extracted from the 1000 Genome project, European_CPIC: European population data extracted from the Clinical PGx Implementation Consortium

**Fig. 2:**
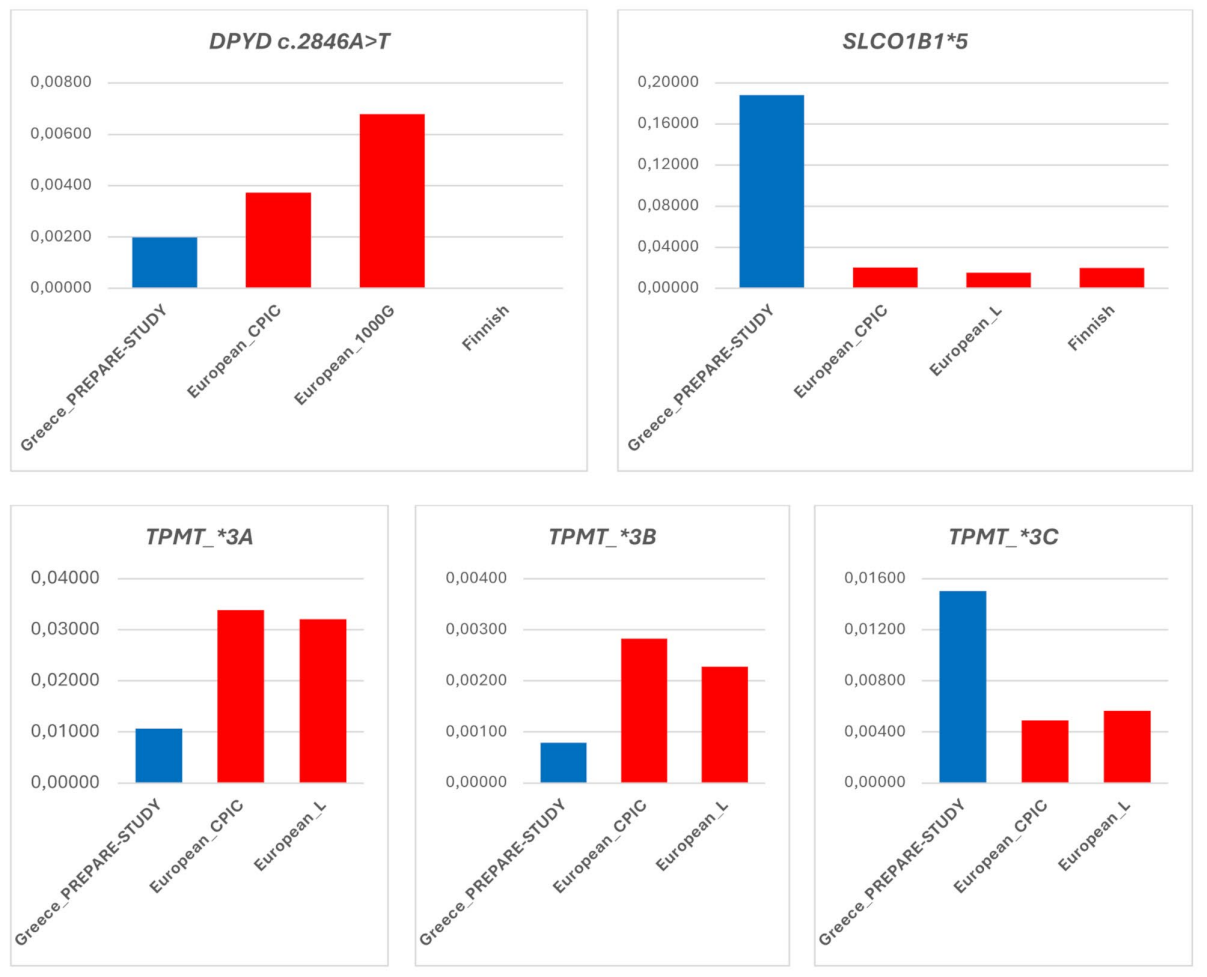
Prevalence of PGx biomarkers in other pharmacogenes in the Greek population (in blue), compared to the European average, as documented in the CPIC database, the 1000 Genomes project, the literature, and the Finnish population (in red). Abbreviations: European_L: European population data extracted from the literature, European_1000G: European population data extracted from the 1000 Genome project, European_CPIC: European population data extracted from the Clinical PGx Implementation Consortium

Our genotyping approach has demonstrated differences in the prevalence of 17 PGx biomarkers in seven pharmacogenes between the Greek population and the European average, deducted from more than one source as summarized in Figs. 1 and 2. In particular, there are four PGx biomarkers in the *CYP2C19* gene, namely CYP2C19*4, CYP2C19*5, CYP2C19*6 and CYP2C19*9, two PGx biomarkers in the *CYP2C9* gene, namely CYP2C9*5 and CYP2C9*11, two PGx biomarkers in the *CYP2B6* gene, namely CYP2B6*4 and CYP2B6*9, two PGx biomarkers in the *CYPD6* gene, namely CYPD6*10 and CYPD6*17, and one PGx biomarker in each one of the *SLCO1B1* and *TPMT* genes, namely SLCO1B1*5 and TPMT*3 C, respectively, that present with higher allelic frequencies (more than 2-fold) compared to the European average from at least two of the available sources. Also, there are two PGx biomarkers in the *TPMT* gene, namely *TPMT* *3A and *TPMT* *3B, and one PGx biomarker in the *CYP2D6*, *CYP2B6*, and *DPYD* genes, namely CYP2D6*5, CYP2B6*5, and *DPYD*:c.2846 A > T, respectively, that present with lower allelic frequencies (more than 2-fold) compared to the European average from at least two of the available sources. Data from the Finnish population and the 1000G project were not available for a few pharmacogenes such as *TPMT*,* SLCO1B1* so there were not represented in Figs. 1 and 2. In those cases, comparisons were made based on CPIC data or available literature for the European population.

**Fig. 3:**
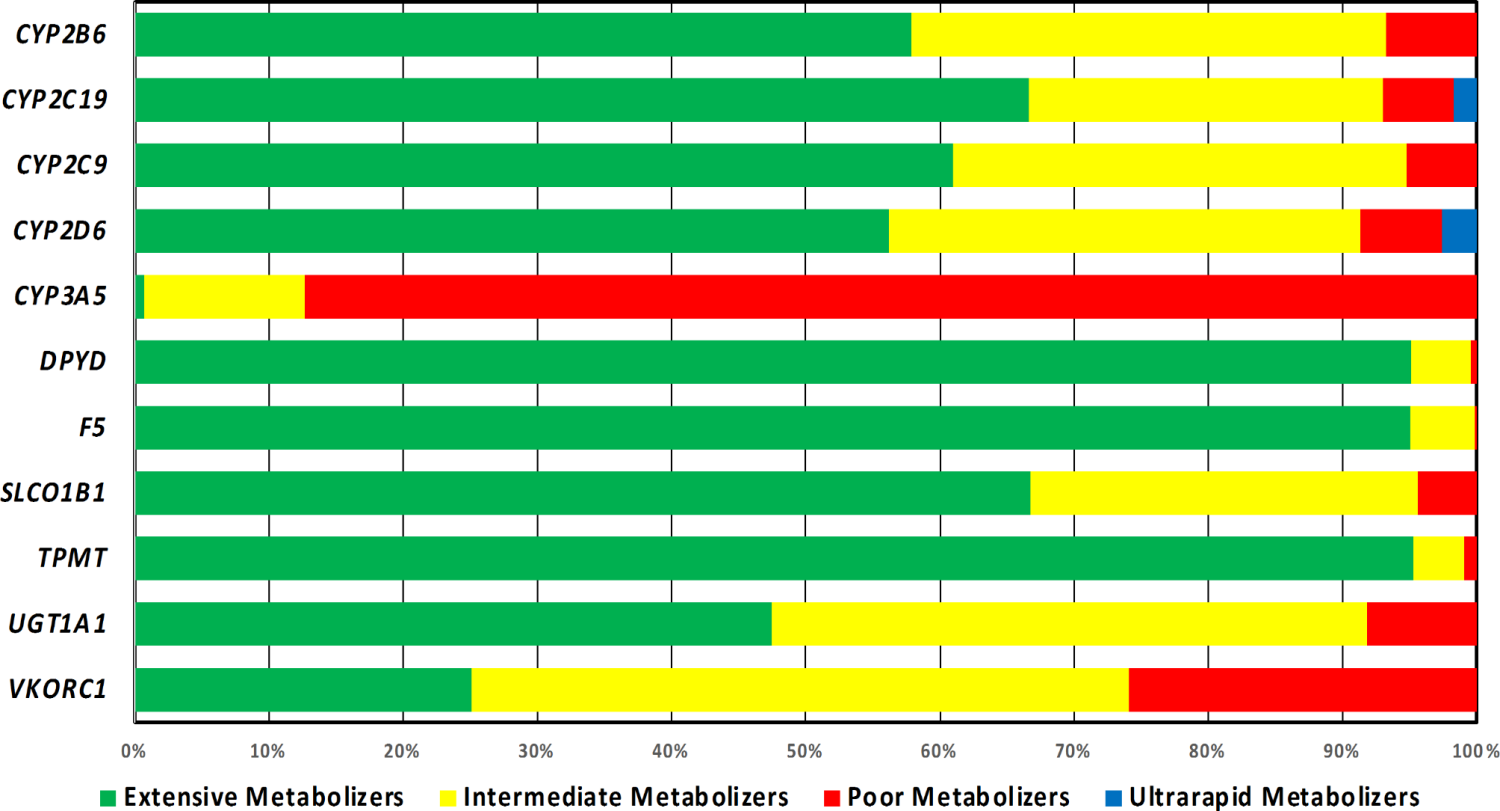
Frequencies of the various metabolizer statuses in the different pharmacogenes

We have also calculated the prevalence of the different metabolizer statuses in the different pharmacogenes analyzed herein (Table 1; Fig. 3). At the gene level, *VKORC1* (74.94%), *UGT1A1* (52.53%) and *CYP2D6* (43.81%) were the top genes with the highest frequency of actionable phenotypes, reflecting on warfarin (*VKORC1*), atazanavir, irinotecan (*UGT1A1*) and most importantly a variety of medications, such as psychiatric, antihypertensive, antiarrhythmic, tamoxifen, codeine, tramadol and others (*CYP2D6*). *CYP3A5*,* TPMT*, and *DPYD* had the lowest frequencies of actionable phenotypes, namely 0.07%, 4.7%, and 4.9% respectively, in line with the expected population prevalence and confirming findings from our previous pilot study [7].

**Table 1:** Frequencies of the various metabolizer statuses in the different pharmacogenes analyzed for the purpose of this study (see also Fig. 3)

Gene	Extensive Metabolizers	Intermediate Metabolizers	Poor Metabolizers	Ultrarapid Metabolizers
***CYP2D6***	0.562	0.351	0.061	0.026
***CYP2C9***	0.610	0.338	0.053	N.A.
***CYP2C19***	0.666	0.264	0.053	0.017
***CYP3A5***	0.007	0.120	0.873	N.A.
***CYP2B6***	0.578	0.353	0.068	N.A.
***VKORC1***	0.251	0.490	0.259	N.A.
***UGT1A1***	0.475	0.443	0.082	N.A.
***DPYD***	0.951	0.044	0.005	N.A.
***TPMT***	0.953	0.038	0.009	N.A.
***SLCO1B1***	0.667	0.288	0.044	N.A.
***F5***	0.950	0.048	0.002	N.A.

### Academic PGx research laboratories and faculty members

Two academic research laboratories bear PGx in their official title. The Laboratory of Pharmacogenomics and Individualized Therapy of the University of Patras, department of Pharmacy (www.permed.upatras.gr) is involved in PGx research using a holistic approach, namely from the wet- and dry-lab and public health genomics perspectives [17]. This laboratory was also involved in the PREemptive Pharmacogenomic Testing for Preventing Adverse Drug Reactions (PREPARE) clinical study (see below). Also, the Laboratory of Molecular Genetics and Pharmacogenomics/Toxicogenomics of the department of Molecular Biology and Genetics, Democritus University of Thrace (DUTH) is involved in PGx research, although none of its faculty members bear PGx in their official academic titles. In addition, the Clinical Genomics and Pharmacogenomics Unit belongs to the 4th Pathology Clinic of the School of Medicine of the National and Kapodistrian University of Athens (NKUA), whose members are also involved in PGx research. There are also a few other academic laboratories that are involved in PGx research activities, but they do not bear PGx as a term in their official titles.

There are four faculty members in Greece that bear PGx in their official academic title and are involved in PGx research; one at the DUTH, Faculty of Medicine, Laboratory of Pharmacology, one at the School of Medicine Clinical Genomics and Pharmacogenomics Unit at NKUA, one at the Aristotle University of Thessaloniki, department of Pharmacy, Thessaloniki and the author’s Institution, namely the University of Patras, department of Pharmacy, Laboratory of Pharmacogenomics and Individualized Therapy, Patras. The author was also a Full member and National Representative of Greece at the European Medicines Agency, CHMP-Pharmacogenomics Working Party from July 2010 until December 2022, at which time the Working Party was retired by the EMA and integrated into the Methodology Working Party.

### PGx education at the undergraduate and postgraduate level in Greece

Our findings from a web-based survey that was conducted in May 2024 have revealed some differences compared to our previous analysis performed in March 2014 [16] as far as PGx education in Greece is concerned.

At the undergraduate level, we have assessed the latest curricula of the Schools/Faculties/Departments of Medicine, Pharmacy, and Biology of all Greek Universities. Of the seven Medical Faculties in Greece, the Faculty of Medicine of the University of Ioannina (UoI) and the one of DUTH have an elective course dedicated to PGx, while all other five Medical Faculties/Schools have sessions dedicated to PGx, as part of other compulsory or elective courses on Pharmacology. From the three departments of Pharmacy, the University of Patras (UPAT) has an compulsory course dedicated to PGx, the Aristotle University of Thessaloniki (AUTH) has an elective course dedicated to PGx, while there is no such dedicated PGx course at the department of NKUAbut only discusses PGx topics as part of the Pharmacology course. Based on our research, there is no compulsory or elective course focused on PGx at the four biology departments of Greek Universities. However, the PGx topic is included in the undergraduate curricula, as part of courses on Human Genetics, Human Molecular Genetics, Genomics, Biochemical Pharmacology, and Clinical and Pharmaceutical Biotechnology in three departments out of the four. More precisely, Molecular Biology and Genetics (DUTH), Biochemistry and Biotechnology (University of Thessaly; UTH), and Biotechnology (Agricultural University of Athens) have a dedicated PGx course. On the contrary, the department of Biology of the University of Crete (UoC) has no course or session dedicated to PGx.

**Fig. 4:**
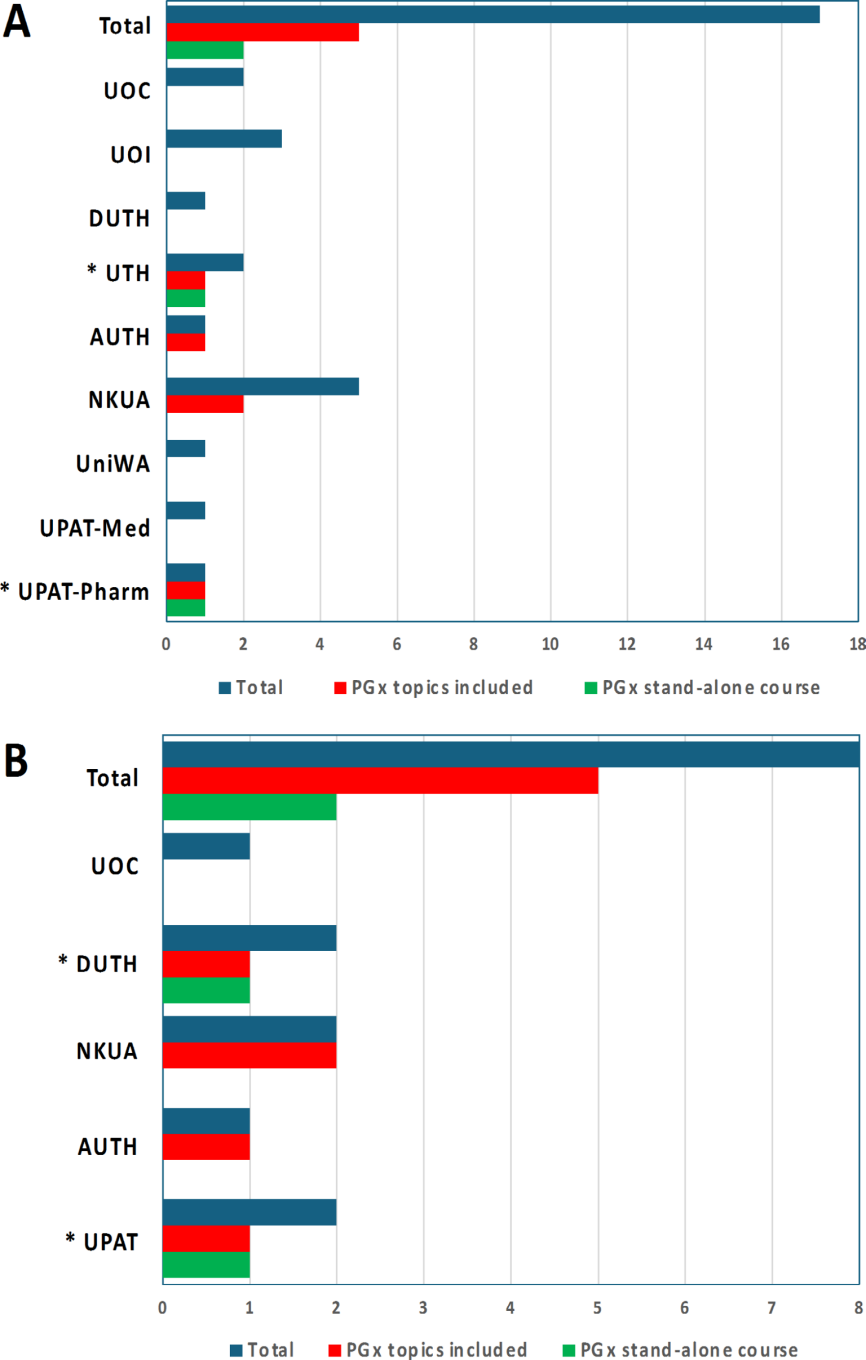
Overview of the departmental (**A**) and interdepartmental/interinstitutional (**B**) postgraduate courses that have stand-alone PGx courses (in green) and/or discuss PGx topics within other related courses in Greek Universities (total number of courses in blue). UOC: University of Crete, UOI: University of Ioannina, DUTH: Democritus University of Thrace, UTH: University of Thessaly, AUTH: Aristotle University of Thessaloniki, NKUA: National and Kapodistrian University of Athens, UniWA: University of Western Attica, UPAT: University of Patras, Med: Department of Medicine, Pharm: Department of Pharmacy. Asterisk depicts universities with stand-alone PGx courses

At the postgraduate level, PGx stand-alone courses and lectures in related courses are available both in departmental and interdepartmental and inter-institutional graduate programs. In the first case, among eight Universities in Greece, only the UPAT and the UTH provides stand-alone PGx courses, while four Universities (in Patras, Athens, Thessaloniki and Thessaly) include PGx topics in related courses in five of their graduate programs (Fig. 4A). As far as interdepartmental and inter-institutional graduate programs are concerned, the UPAT and DUTH provide stand-alone PGx courses in their interdepartmental graduate programs on Personalized Medicine, while four Universities in Patras, Athens, Thessaloniki and Thrace discuss PGx topics in related courses in 5 of their interdepartmental and inter-institutional graduate programs (Fig. 4B). The University of Crete does not have any stand-alone PGx course or PGx lecture in its three related graduate programs.

### Clinical implementation studies on genome-guided therapeutics

Two multinational, multicenter, prospective PGx clinical implementation studies have been performed in Greece. The European Pharmacogenetics for AntiCoagulant Therapy project (EU-PACT; NCT01119300, https://clinicaltrials.gov/ct2/show/NCT01119300) included two single-blind, randomized trials that aimed to compare a genotype-guided dosing algorithm that included clinical variables and genotyping for *CYP2C9* and *VKORC1* with a dosing algorithm that included only clinical variables, for the initiation of warfarin, acenocoumarol or phenprocoumon treatment in patients with atrial fibrillation or venous thromboembolism [18, 19]. The primary outcome was the percentage of time in the target range for the international normalized ratio (INR; target range, 2.0 to 3.0) within a 12-week period after the initiation of therapy. In the clinical study, four countries were involved in patient recruitment, namely the UK (warfarin, acenocoumarol, phenprocoumon), Sweden (warfarin), Greece (acenocoumarol), and the Netherlands (acenocoumarol, phenprocoumon). In Greece, a total of 207 patients were recruited from three sites across Greece; the University General Hospital of Alexandroupolis, Democritus University of Thrace, Faculty of Medicine, and the Onassis Cardiac Surgery Center in Athens, Greece. The EU-PACT study concluded that PGx-guided dosing of warfarin was associated with a higher percentage of time in the therapeutic INR range compared to standard care during the initiation of warfarin therapy, whereas PGx-guided dosing of acenocoumarol or phenprocoumon didn’t show any relevant improvement during the 12 weeks after the initiation of therapy.

Moreover, a second study, namely the PREemptive Pharmacogenomic testing for Preventing Adverse Drug Reactions study (PREPARE; NCT03093818; https://classic.clinicaltrials.gov/ct2/show/NCT03093818) was an open-label, multicentre, controlled, cluster-randomized, crossover implementation study of a 12-gene pharmacogenetic panel in 18 hospitals, nine community health centers, and 28 community pharmacies across seven European countries (Austria, Greece, Italy, the Netherlands, Slovenia, Spain, and the UK) [13]. Having recruited a total of 6944 patients with primary indications spanning several medical specialties including oncology, cardiology, psychiatry, and so on, it was shown that genome-guided treatment using a 12-gene PGx panel significantly reduced the incidence of clinically relevant adverse drug reactions and was feasible across diverse European health-care system organisations and settings. In Greece, 1326 psychiatric patients were recruited in total at two sites; the University General Hospital of Patras and the ATTIKON University General Hospital in Athens, Greece [20].

### Private and public PGx service providers in Greece

Our data show that there are nine out of 21 private genetic laboratories in Greece that offer PGx testing services (41%). Also, there is only one public (academic) PGx laboratory that offers PGx testing services that is also accredited for PGx services by the European Molecular Diagnostics Quality Network. Most of these offer PGx testing services for various medical specialties and/or medications, while some of them specialize in specific medical specialties such as oncology or psychiatry.

It seems that no new private or public laboratories have incorporated PGx services in their flow. According to our previous studies, the number of genetic laboratories that offer PGx testing services remains more or less unaltered (41% compared to 44% in 2011; 15), even though few of them have discontinued their operations, while others were founded. This observation does not give a clear picture of the actual demand for this type of services in the market. In other words, it is unclear whether there is a significant interest of healthcare professionals to prescribe such testing even though there are specialized laboratories who offer them. It must be noted that only a couple of PGx tests for oncology and, recently, for psychiatry are being reimbursed by the public payer. The latter test, however, also includes genomic variants that are not clinically actionable, which questions the clinical validity of this test, as the result provided to the patient/treating physician is not fully approved by the EMA or any other regulatory body worldwide.

### Regulatory guidance for genome-guided drug dose adjustment in Greece

We have previously revealed a vast discrepancy among the regulatory guidance for PGx-guided treatment among the different regulatory bodies worldwide [21, 22]. As such, this has prompted us to inquire whether such discrepancy also exists between the Greek NOM and its supervisory body, namely the EMA and the Galinos Greek medicines repository.

**Fig. 5:**
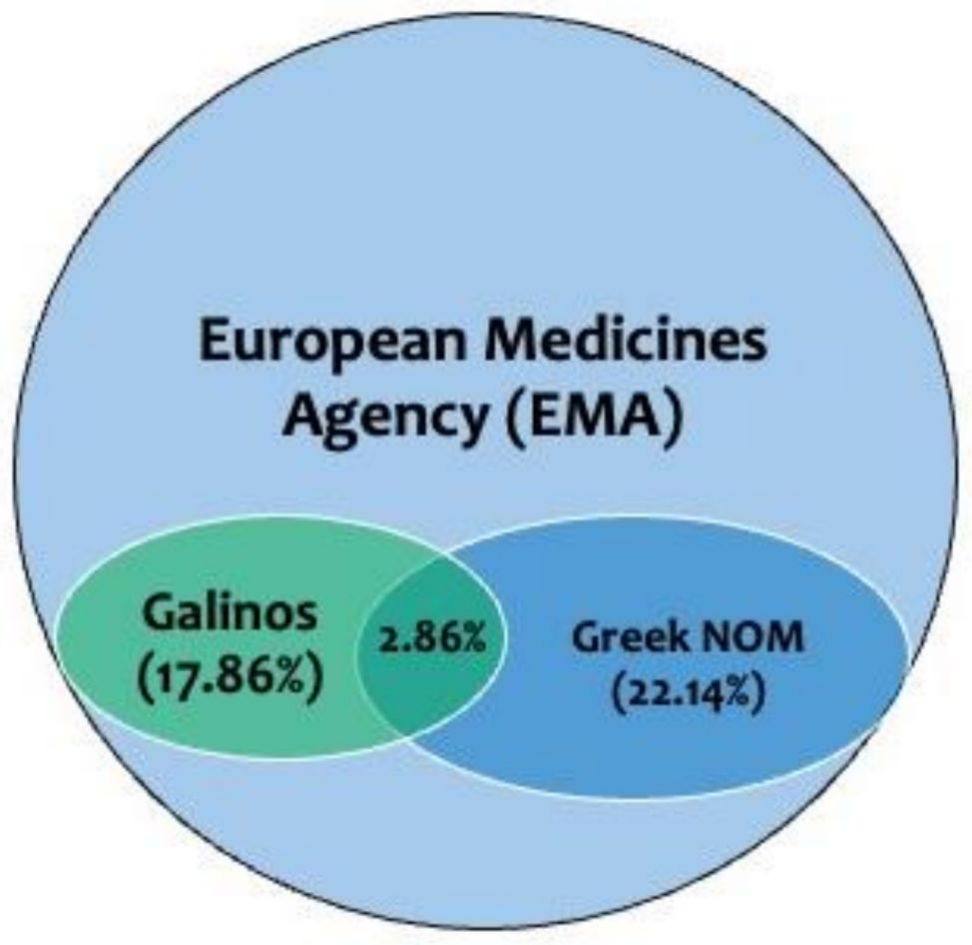
VENN diagram indicating the vast discrepancy between the medications that have PGx guidance for drug dose adjustment in the SmPCs as documented in the Greek NOM and Galinos, compared to the EMA (see also text for details)

Interestingly though, there is a vast discrepancy between the medications that have PGx guidance for drug dose adjustment in the SmPCs as documented in the Greek NOM and Galinos, compared to the EMA. In particular, there are 140 active compounds available in the EMA drug annotations list, as shown in the PharmGKB database. From these, there are only 31 active compounds that are documented in the Greek NOM and the EMA (22.14%), while only 25 active compounds are listed in Galinos and the EMA (17.86%). Moreover, only four active compounds are documented in the Galinos and the Greek NOM (2.86%; Fig. 5). Evidently, there is a big difference between the number of active compounds listed in the Greek NOM and Galinos per se, and the indications that these medications refer to. In particular, 28 out of 31 active compounds in the Greek NOM refer to anticancer medications and three out of 31 refer to medications for diabetes, while the 25 active compounds listed in the Galinos resource were more diverse since they belong to five anticancer medications, four antiviral, three psychiatric medications, two medications for the urinary system and 11 for other indications. Three of four active compounds that are present both in the Greek NOM and the Galinos are anticancer medications, namely irinotecan (*UGT1A1*), capecitabine (*DPYD*), and erlotinib (*EGFR*), while one is an antidiabetic medication namely glibenclamide (*G6PD*). It is noteworthy that although the Greek NOM is a formal member of the EMA, only 22.14% of the medications with approved PGx information in their labels by the EMA are listed.

## Discussion

In recent years, PGx has gradually assumed an integral part in modern healthcare, enjoying broad application in several medical specialties. However, for PGx to be properly integrated into mainstream clinical practice, a multifaceted approach and a well-orchestrated effort must be undertaken that includes capacity building for PGx testing services, genomics education of healthcare professionals, cost-effectiveness analysis to ensure that such services can be reimbursed by the payers and the establishment of the proper legal framework to ensure that the necessary ethical and societal aspects of this modern discipline are adequately addressed. As such, it is not a surprise that the degree of clinical implementation of PGx in different countries is very heterogeneous with the United States and countries in Northwestern Europe, such as the United Kingdom and the Netherlands to pave the path. Unfortunately, the pace of clinical implementation in other European countries is not so advanced, especially in Southern European countries.

In this article, we report our findings from a systematic analysis of the PGx landscape in Greece to document the current state of PGx in the country, including the level of PGx education in Greek academic institutions, the availability of PGx testing services in private and public genetic laboratories and the regulatory discrepancies in Greece. We have also paid particular attention to determining the prevalence of PGx biomarkers in the Greek population, which shows differences compared to the European average, as documented in various sources and genetic databases.

Genotyping analysis of the Greek population reveals that there are many more PGx variants whose prevalence is substantially different to the European average. This finding agrees with our previous pilot study [7] and it overcomes it. This is expected, since the number of individuals analyzed was also significantly more (1269 individuals in this study versus 44 in [7]), underlying the need to include larger and more representative cohorts to determine the PGx biomarkers molecular spectrum. Thanks to the aforementioned analysis, the current PGx biomarker spectrum in Greece spans across many medical specialties, such as oncology (*CYP2D6*, *TPMT*, *DPYD*), psychiatry (*CYP2D6*, *CYP2C19*), cardiology (*CYP2D6*, *CYP2C9*, *VKORC1*, *SLCO1B1*), and infectious diseases (*CYP2B6*) [14]. Since all the above PGx biomarkers are clinically actionable residing within very important pharmacogenes, these findings can guide important public health PGx decisions to adopt specific policies at the population level, underlining the importance of population PGx in the clinical application of precision therapeutics [23]. At the same time, these data confirm that PGx biomarkers allele frequency data from racial groups are simply not sufficient for extrapolation at a population level, since there are significant differences among populations belonging to the same racial group [24].

A crucial aspect of the clinical implementation of PGx is educating healthcare professionals in PGx and personalized medicine. Our data from scanning the PGx educational environment in Greek academic institutions show that PGx is extensively taught in the undergraduate and postgraduate curricula. This situation is significantly improved compared to our previous analysis in 2014 [16], as only one out of 17 departments in 8 Universities in Greece (5.9%) does neither have a stand-alone PGx course nor discuss PGx topics in relevant courses (data available upon request). This is directly reflected in our recent findings, indicating that most healthcare professionals are aware of the notion of genome-guided therapeutics [25].

Our findings also highlight a significant bottleneck in the clinical implementation of PGx in Greece, which touches upon regulatory and reimbursement aspects. First of all, our data show that there is a significant lack of PGx information in the drug labels (only 31 active compounds are documented in the Greek NOM out of 140 that are annotated in the EMA, its supervising regulatory body). At the same time, there are 25 active compound annotations in the Galinos drug repository, from which only four are also present in the Greek NOM list. This can create a confusion among healthcare professionals in Greece, who are not only confronted with a substantially shorter list of drug/gene annotations compared to the EMA’s list but also a vastly heterogeneous list of drug/gene annotations between the two drug repositories in Greece. These discrepancy calls for an immediate rectification action, led by the official Greek NOM authority to address this bottleneck. At the same time, only a very small percentage of PGx tests are reimbursed by the Greek payers, which significantly hampers the smooth integration of PGx in mainstream healthcare. In addition, one type of reimbursed PGx testing includes PGx biomarkers with pharmacogenes that are not clinically actionable and, hence without any credible and regulatory-approved guidance for drug dose optimization. This can raise significant bioethical issues as far as patient safety is concerned. Lastly, the lack of accreditation for the various PGx testing laboratories is yet another issue that warrants close regulatory monitoring, especially when it comes to the very few PGx tests that are reimbursed by the payers in Greece.

### Limitations

This study has a few limitations. As far as the search for laboratory services in PGx, the study was limited since no direct interviews or surveys were done to get concise feedback on the services offered versus the services sold. Thus, there is no exact metric for the existing demand for PGx tests in the Greek healthcare sector. Moreover, there are only two main PGx tests that got recently approved for reimbursement by the Greek healthcare fund, so the data on the demand are still limited.

## Conclusions

In this study, we demonstrate that during the past ten years, there have been significant advances in the field of PGx in Greece. Data from prospective clinical studies have become available, indicating that precision therapeutics can lead to significant improvements in drug safety in psychiatric patients, as well as significant cost savings for the healthcare system in Greece [20] and other European countries [26–28]. Also, many PGx testing laboratories can provide PGx testing services and healthcare professionals seem to be sufficiently aware of the benefits of PGx testing. The PGx educational environment has been further harmonized and the biomedical curricula have been significantly enriched with PGx topics within stand-alone or related PGx courses. There are still a few but important hurdles of regulatory and economic nature, that need to be overcome, which also have significant ethical implications.

The next step to broadly introduce PGx testing in the Greek healthcare setting requires a strong political will to move ahead with those medical specialties for which clinical and economic evidence on the benefit of PGx testing exists, such as psychiatry [20] and at the same time rectify the few existing bottlenecks, as outlined above. Incentives should also be given to established PGx research groups in Greece to provide additional clinical and economic evidence for PGx-guided interventions in other medical specialties, possibly as public-private partnerships, that would enrich the battery of PGx-guided interventions in Greece. It would be also important to integrate results from PGx testing results into electronic medical records via intelligence technology applications to increase the available clinical evidence and get insight into the results of large-scale genomics projects, such as the Genome of Greece (www.gogreece.org.gr). Finally, introducing a unified and standardized accreditation and validation process for PGx laboratories would improve the credibility of PGx results and increase the demand for such services.

## Data Availability

Data are provided within the manuscript and are also available upon request.

## References

[1] Squassina A, Manchia M, Manolopoulos VG, Artac M, Lappa-Manakou C, Karkabouna S, et al. Realities and expectations of pharmacogenomics and personalized medicine: impact of translating genetic knowledge into clinical practice. Pharmacogenomics. 2010;11(8):1149–67. 20712531 10.2217/pgs.10.97

[2] Davies EC, Green CF, Mottram DR, Pirmohamed M. Interpreting adverse drug reaction (ADR) reports as hospital patient safety incidents. Br J Clin Pharmacol. 2010;70(1):102–8. 20642552 10.1111/j.1365-2125.2010.03671.x PMC2909812

[3] Zineh I, Pacanowski MA. Pharmacogenomics in the assessment of therapeutic risks versus benefits: inside the United States Food and Drug Administration. Pharmacotherapy. 2011;31(8):729–35. 21923598 10.1592/phco.31.8.729

[4] Ehmann F, Caneva L, Prasad K, Paulmichl M, Maliepaard M, Llerena A, et al. Pharmacogenomic information in drug labels: European Medicines Agency perspective. Pharmacogenomics J. 2015;15(3):201–10. 25707393 10.1038/tpj.2014.86

[5] Arnold FL, Fukunaga S, Kusama M, Matsuki N, Ono S. Assessment of factors associated with dose differences between Japan and the United States. Clin Pharmacol Ther. 2014;95(5):542–9. 24281222 10.1038/clpt.2013.231

[6] Wilson JF, Weale ME, Smith AC, Gratrix F, Fletcher B, Thomas MG, et al. Population genetic structure of variable drug response. Nat Genet. 2001;29(3):265–9. 11685208 10.1038/ng761

[7] Mizzi C, Dalabira E, Kumuthini J, Dzimiri N, Balogh I, Başak N, Böhm R, Borg J, Borgiani P, Bozina N, Bruckmueller H, Burzynska B, Carracedo A, Cascorbi I, Deltas C, Dolzan V, Fenech A, Grech G, Kasiulevicius V, Kádaši Ľ, Kučinskas V, Khusnutdinova E, Loukas YL, Macek M Jr, Makukh H, Mathijssen R, Mitropoulos K, Mitropoulou C, Novelli G, Papantoni I, Pavlovic S, Saglio G, Setric J, Stojiljkovic M, Stubbs AP, Squassina A, Torres M, Turnovec M, van Schaik RH, Voskarides K, Wakil SM, Werk A, Del Zompo M, Zukic B, Katsila T, Lee MT, Motsinger-Rief A, Mc Leod HL, van der Spek PJ, Patrinos GP. A European spectrum of pharmacogenomic biomarkers: implications for clinical pharmacogenomics. PLoS ONE. 2016;11(9):e0162866. 27636550 10.1371/journal.pone.0162866 PMC5026342

[8] Runcharoen C, Fukunaga K, Sensorn I, Iemwimangsa N, Klumsathian S, Tong H, Vo NS, Le L, Hlaing TM, Thant M, Zain SM, Mohamed Z, Pung YF, Capule F, Nevado J Jr, Silao CL, Al-Mahayri ZN, Ali BR, Yuliwulandari R, Prayuni K, Zahroh H, Noor DAM, Xangsayarath P, Xayavong D, Kounnavong S, Sayasone S, Kordou Z, Liopetas I, Tsikrika A, Tsermpini EE, Koromina M, Mitropoulou C, Patrinos GP, Kesornsit A, Charoenyingwattana A, Wattanapokayakit S, Mahasirimongkol S, Mushiroda T, Chantratita W. Prevalence of pharmacogenomic variants in 100 pharmacogenes among southeast Asian populations under the collaboration of the Southeast Asian Pharmacogenomics Research Network (SEAPharm). Hum Genome Var. 2021;8(1):7. 33542200 10.1038/s41439-021-00135-z PMC7862625

[9] Al-Mahayri ZN, Patrinos GP, Wattanapokayakit S, Iemwimangsa N, Fukunaga K, Mushiroda T, Chantratita W, Ali BR. Variation in 100 relevant pharmacogenes among emiratis with insights from understudied populations. Sci Rep. 2020;10(1):21310. 33277594 10.1038/s41598-020-78231-3 PMC7718919

[10] Bonifaz-Peña V, Contreras AV, Struchiner CJ, Roela RA, Furuya-Mazzotti TK, Chammas R, et al. Exploring the distribution of genetic markers of pharmacogenomics relevance in Brazilian and Mexican populations. PLoS ONE. 2014;9(11):e112640. 25419701 10.1371/journal.pone.0112640 PMC4242606

[11] Kampourakis K, Vayena E, Mitropoulou C, Borg J, van Schaik RH, Cooper DN, et al. Key challenges for next next-generation pharmacogenomics. EMBO Rep. 2014;15(5):472–6. 24723683 10.1002/embr.201438641 PMC4210086

[12] Patrinos GP, Pasparakis E, Koiliari E, Pereira AC, Hünemeier T, Pereira LV, Mitropoulou C. Roadmap for establishing large-scale genomic medicine initiatives in low- and middle-income countries. Am J Hum Genet. 2020;107(4):589–95. 33007198 10.1016/j.ajhg.2020.08.005 PMC7536572

[13] Swen JJ, van der Wouden CH, Manson LE, Abdullah-Koolmees H, Blagec K, Blagus T, Böhringer S, Cambon-Thomsen A, Cecchin E, Cheung KC, Deneer VH, Dupui M, Ingelman-Sundberg M, Jonsson S, Joefield-Roka C, Just KS, Karlsson MO, Konta L, Koopmann R, Kriek M, Lehr T, Mitropoulou C, Rial-Sebbag E, Rollinson V, Roncato R, Samwald M, Schaeffeler E, Skokou M, Schwab M, Steinberger D, Stingl JC, Tremmel R, Turner RM, van Rhenen MH, Dávila Fajardo CL, Dolžan V, Patrinos GP, Pirmohamed M, Sunder-Plassmann G, Toffoli G, Guchelaar HJ, Ubiquitous Pharmacogenomics Consortium. A 12-gene pharmacogenetic panel to prevent adverse drug reactions: an open-label, multicentre, controlled, cluster-randomised crossover implementation study. Lancet. 2023;401(10374):347–56. 36739136 10.1016/S0140-6736(22)01841-4

[14] van der Wouden CH, Cambon-Thomsen A, Cecchin E, Cheung KC, Dávila-Fajardo CL, Deneer VH, Dolžan V, Ingelman-Sundberg M, Jönsson S, Karlsson MO, Kriek M, Mitropoulou C, Patrinos GP, Pirmohamed M, Samwald M, Schaeffeler E, Schwab M, Steinberger D, Stingl J, Sunder-Plassmann G, Toffoli G, Turner RM, van Rhenen MH, Swen JJ, Guchelaar HJ, Ubiquitous Pharmacogenomics Consortium. Implementing pharmacogenomics in Europe: design and implementation strategy of the ubiquitous Pharmacogenomics Consortium. Clin Pharmacol Ther. 2017;101(3):341–58. 28027596 10.1002/cpt.602

[15] Sagia A, Cooper DN, Poulas K, Stathakopoulos V, Patrinos GP. A critical appraisal of the private genetic and pharmacogenomic testing environment in Greece. Per Med. 2011;8(4):413–20. 29783335 10.2217/pme.11.24

[16] Pisanu C, Tsermpini EE, Mavroidi E, Katsila T, Patrinos GP, Squassina A. Assessment of the Pharmacogenomics Educational Environment in Southeast Europe. Public Health Genomics. 2014;17(5–6):272–9. 25341999 10.1159/000366461

[17] Patrinos GP, Katsila T. Pharmacogenomics education and research at the Department of Pharmacy, University of Patras. Greece Pharmacogenomics. 2016;17(17):1865–72. 27790924 10.2217/pgs-2016-0142

[18] Pirmohamed M, Burnside G, Eriksson N, Jorgensen AL, Toh CH, Nicholson T, Kesteven P, Christersson C, Wahlström B, Stafberg C, Zhang JE, Leathart JB, Kohnke H, van der Maitland- AH, Williamson PR, Daly AK, Avery P, Kamali F, Wadelius M. EU-PACT Group. A randomized trial of genotype-guided dosing of warfarin. N Engl J Med. 2013;369(24):2294–303. 24251363 10.1056/NEJMoa1311386

[19] Verhoef TI, Ragia G, de Boer A, Barallon R, Kolovou G, Kolovou V, Konstantinides S, Le Cessie S, Maltezos E, van der Meer FJ, Redekop WK, Remkes M, Rosendaal FR, van Schie RM, Tavridou A, Tziakas D, Wadelius M, Manolopoulos VG. Maitland-Van Der Zee AH; EU-PACT Group. A randomized trial of genotype-guided dosing of acenocoumarol and phenprocoumon. N Engl J Med. 2013;369(24):2304–12. 24251360 10.1056/NEJMoa1311388

[20] Skokou M, Karamperis K, Koufaki MI, Tsermpini EE, Pandi MT, Siamoglou S, Ferentinos P, Bartsakoulia M, Katsila T, Mitropoulou C, Patrinos GP. Consortium of the PREPARE study in Greece. Clinical implementation of preemptive pharmacogenomics in psychiatry. EBioMedicine. 2024;101:105009. 38364700 10.1016/j.ebiom.2024.105009 PMC10879811

[21] Koutsilieri S, Tzioufa F, Sismanoglou DC, Patrinos GP. Unveiling the guidance heterogeneity for genome-informed drug treatment interventions among regulatory bodies and research consortia. Pharmacol Res. 2020;153:104590. 31830522 10.1016/j.phrs.2019.104590

[22] Koutsilieri S, Tzioufa F, Sismanoglou DC, Patrinos GP. Towards harmonizing guidance for genome-informed drug treatment interventions: the show must go on. Pharmacol Res. 2020;158:104839. 32438033 10.1016/j.phrs.2020.104839

[23] Mette L, Mitropoulos K, Vozikis A, Patrinos GP. Pharmacogenomics and public health: implementing ‘populationalized’ medicine. Pharmacogenomics. 2012;13(7):803–13. 22594512 10.2217/pgs.12.52

[24] Karamperis K, Katz S, Melograna F, Ganau FP, Van Steen K, Patrinos GP, Lao O. A worldwide spectrum of pharmacogenomic variants associated with drug-related toxic events: Genetic structure and risk probability in population sequencing data unravel geographical patterns among Eastern and Western populations. iScience. 2024, in press. 10.1016/j.isci.2024.110916 PMC11465127 39391720

[25] Koufaki MI, Patrinos GP, Vasileiou KZ. A qualitative approach to assess the opinion of physicians about the challenges and prospects of pharmacogenomic testing implementation in clinical practice in Greece. Hum Genomics. 2024;18(1):82. 39030587 10.1186/s40246-024-00648-y PMC11264745

[26] Fragoulakis V, Roncato R, Bignucolo A, Patrinos GP, Toffoli G, Cecchin E, Mitropoulou C. Cost-utility analysis and cross-country comparison of pharmacogenomics-guided treatment in colorectal cancer patients participating in the U-PGx PREPARE study. Pharmacol Res. 2023;197:106949. 37802427 10.1016/j.phrs.2023.106949

[27] Koufaki MI, Fragoulakis V, Díaz-Villamarín X, Karamperis K, Vozikis A, Swen JJ, Dávila-Fajardo CL, Vasileiou KZ, Patrinos GP, Mitropoulou C. Economic evaluation of pharmacogenomic-guided antiplatelet treatment in Spanish patients suffering from acute coronary syndrome participating in the U-PGx PREPARE study. Hum Genomics. 2023;17(1):51. 37287029 10.1186/s40246-023-00495-3 PMC10249170

[28] Fragoulakis V, Koufaki MI, Joefield-Roka C, Sunder-Plassmann G, Mitropoulou C. Cost-utility analysis of pharmacogenomics-guided tacrolimus treatment in Austrian kidney transplant recipients participating in the U-PGx PREPARE study. Pharmacogenomics J. 2024;24(2):10. 38499549 10.1038/s41397-024-00330-5

